# Magnetized plasma implosion in a snail target driven by a moderate-intensity laser pulse

**DOI:** 10.1038/s41598-018-36176-8

**Published:** 2018-12-17

**Authors:** T. Pisarczyk, S. Yu Gus’kov, A. Zaras-Szydłowska, R. Dudzak, O. Renner, T. Chodukowski, J. Dostal, Z. Rusiniak, T. Burian, N. Borisenko, M. Rosinski, M. Krupka, P. Parys, D. Klir, J. Cikhardt, K. Rezac, J. Krasa, Y.-J. Rhee, P. Kubes, S. Singh, S. Borodziuk, M. Krus, L. Juha, K. Jungwirth, J. Hrebicek, T. Medrik, J. Golasowski, M. Pfeifer, J. Skala, P. Pisarczyk, Ph. Korneev

**Affiliations:** 1 Institute of Plasma Physics and Laser Microfusion, Warsaw, Poland; 2 P.N. Lebedev Physical Institute of RAS, Moscow, Russian Federation; 3 National Research Nuclear University MEPhI, Moscow, Russian Federation; 4 Institute of Physics, Czech Academy of Sciences, 182 21 Prague, Czech Republic; 5 Insitute of Plasma Physics, Czech Academy of Sciences, 182 00 Prague, Czech Republic; 6 Faculty of Electrical Engineering, Czech Technical University, 166 27 Prague, Czech Republic; 7 Center for Relativistic Laser Science, IBS, Gwang-Ju, 61005 Korea; 8 Warsaw University of Technology, ICS, Warsaw, Poland

## Abstract

Optical generation of compact magnetized plasma structures is studied in the moderate intensity domain. A sub-ns laser beam irradiated snail-shaped targets with the intensity of about 10^16^ W/cm^2^. With a neat optical diagnostics, a sub-megagauss magnetized plasmoid is traced inside the target. On the observed hydrodynamic time scale, the hot plasma formation achieves a theta-pinch-like density and magnetic field distribution, which implodes into the target interior. This simple and elegant plasma magnetization scheme in the moderate-intensity domain is of particular interest for fundamental astrophysical-related studies and for development of future technologies.

## Introduction

**Figure 1:**
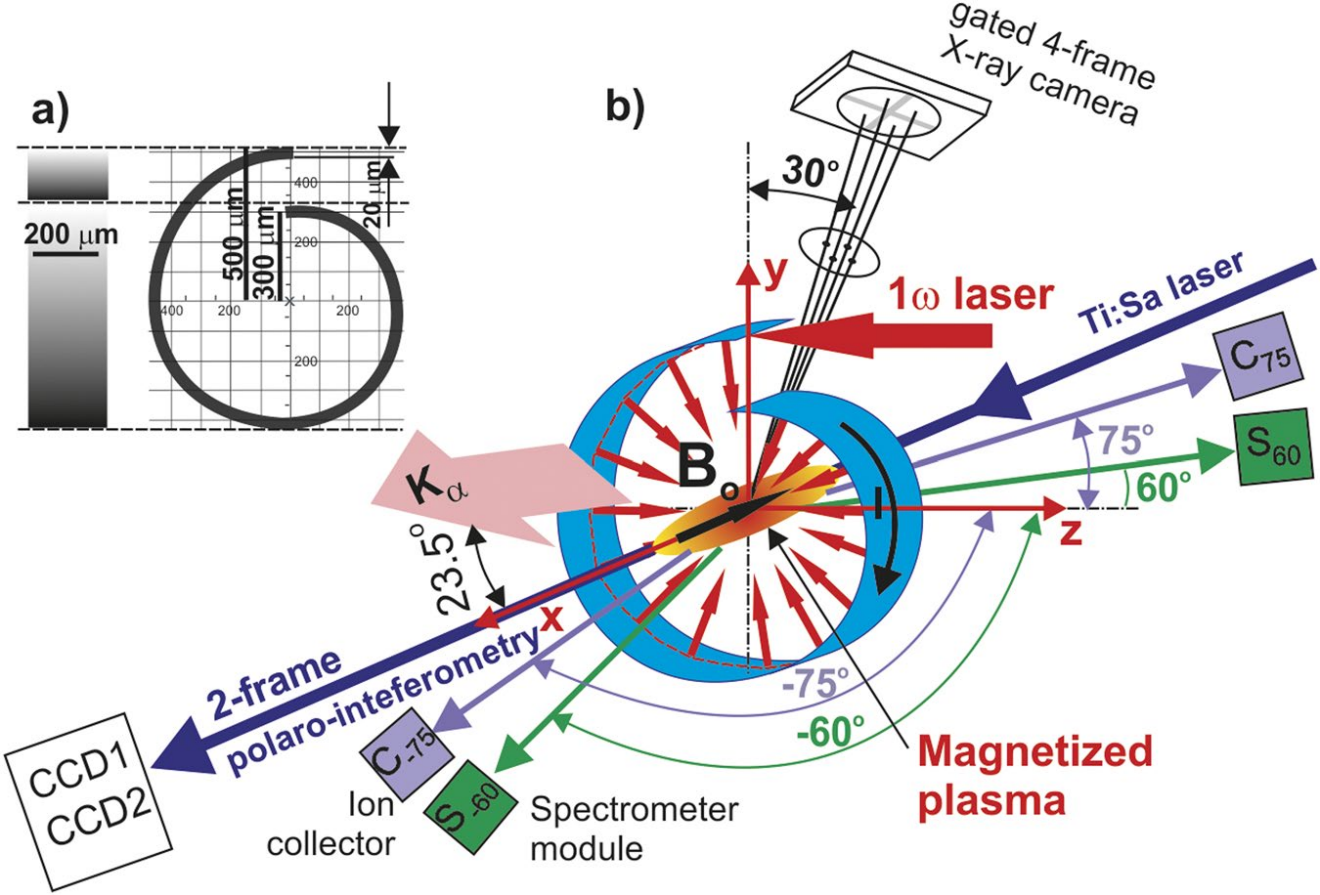
Geometry of the experimental setup: (**a**) the snail target geometry and (**b**) positioning of diverse diagnostics and definition of the driver and diagnostic laser beams propagation. Electron spectrometers and ion collectors are positioned symmetrically (left and right) to the z-axis which is parallel to the main laser beam (in the horizontal plane).

Laboratory astrophysics attracts more and more attention due to progress in laser systems, diagnostics, and magnetic field generation. The latter is very important for modeling astrophysical phenomena, as they mostly deal with magnetized plasma in the universe. Modern coil-based magnetic field generators can produce magnetic fields up to few tens of Tesla, pulsing with microsecond duration in a vacuum chamber^1^. Another approach to magnetic field generation represent optical generators based on the capacitor-coil schemes^2^. However if the magnetic flux is generated in a vacuum, one of the main difficulties for the astrophysical related setups is the magnetization of hot plasmas. In this context, several approaches based on the controllable generation of spontaneous magnetic fields were proposed. Some of them use the Biermann-Battery^3^ or kinetic effects^4,5^ developed under laser irradiation of flat targets, while the others use shaped targets^6,7^. Among these, the snail-shaped targets were subjects of parametrical numerical^8^ and experimental studies^9,10^ in the relativistic intensity domain. In spite of this, no theoretical or experimental results were ever presented for such targets illuminated by moderate-intensity laser pulses. This regime is however extremely interesting since it allows depositing much more energy to the target within a long laser pulse while the resulting plasma structure may appear to be more equilibrium and stable. At the same time, reliable simulations in this interaction domain are tricky as they require an accurate treatment of both hydrodynamic and kinetic processes. In this paper, we present the first experimental study of snail-shaped targets, the geometry of which is schematically shown in Fig. 1a), irradiated by a sub-ns laser beam in the moderate (~10^16^ W/cm^2^) intensity regime.

The interaction process was studied in detail by using primarily an optical diagnostics based on the 2-frame femtosecond polaro-interferometry^5,11^. This method provides a possibility to obtain in a single shot two frames with data containing information on both plasma density and the magnetic field distribution. Additional data was provided by the X-ray K_α_ imaging^12^, electron spectrometer^13^, ion grid collectors^14^, current probe^15^, and a 4-frame X-ray pinhole camera^16^.

The geometry of the experimental setup and laser-target interaction is shown in Fig. 1. The snail-shaped targets with a diameter up to 1 mm, 200 μm length, and 20-μm-thick wall, were manufactured in IPPLM, Warsaw, and LPI RAS, Moscow. As the driver, the linearly (vertically) polarized beam of the PALS iodine laser with the fundamental wavelength of 1315 nm, the FWHM pulse duration of about 350 ps and energy up to 700 J was used. The laser beam was focused to the focal spot of ~100 μm in diameter, thus providing the intensity on the target up to 2·10^16^ W/cm^2^. An auxiliary synchronized Ti:Sa laser pulse^17^ with a wavelength of 808 nm and the pulse duration 40 fs was used for the complex polaro-interferometric diagnostics.

## Experimental Results

### Electron density and magnetic fields

The used polaro-interferometric system represents an advanced version of the single-frame polaro-interferometer^11^. It consists of two independent tracks operating in the complex interferometry regime^18^. In this regime, interferogram fringes relate to the electron density while their intensity modulation corresponds to the Faraday effect in the plasma-frozen magnetic field.

To visualize the process of the plasma formation inside the snail irradiated by the main laser beam, two frames with a time interval of 400 ps were recorded in each shot. The delay between the first frame and the laser intensity maximum varied from −1000 ps to a few ns.

**Figure 2:**
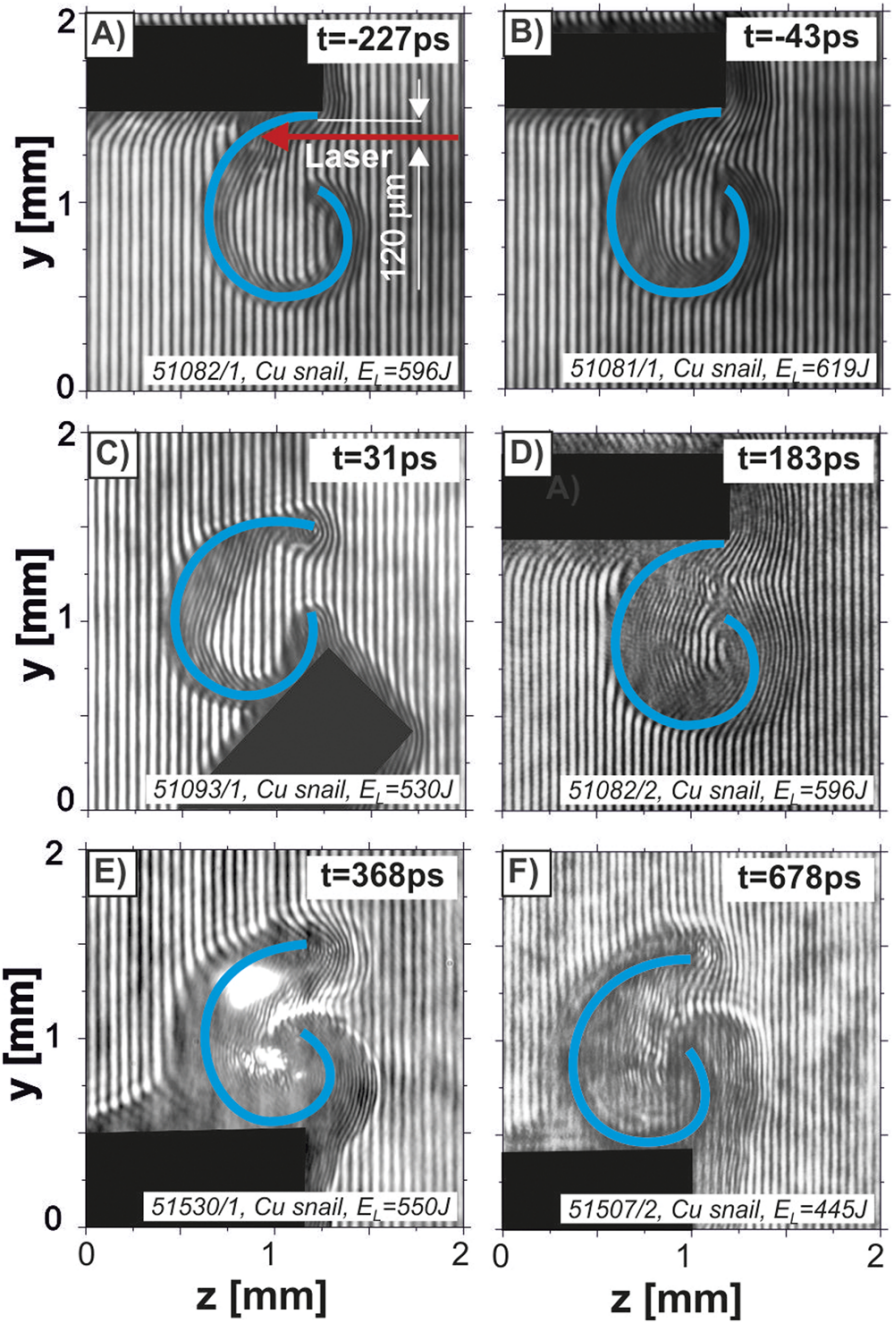
The temporal sequence (−227 ps, −43 ps, 31 ps, 183 ps, 368 ps, 678 ps) of the raw complex interferograms taken by using the laser-driven snail-shaped targets.

To provide the same irradiation conditions for all snail targets, they were illuminated as schematically shown in Fig. 2A. However, random alignment issues might result in ~50 μm deviation from the desired focal position. Different supporting schemes shown in the figure did not affect considerably the process of the magnetized plasma formation in the target. This indicates that the plasma magnetization process is a local effect governed by the local discharges in the interaction region.

Examples of raw complex interferograms taken in a sequence of {−227 ps, −43 ps, 31 ps, 183 ps, 368 ps, 678 ps} are presented in Fig. 2.

**Figure 3:**
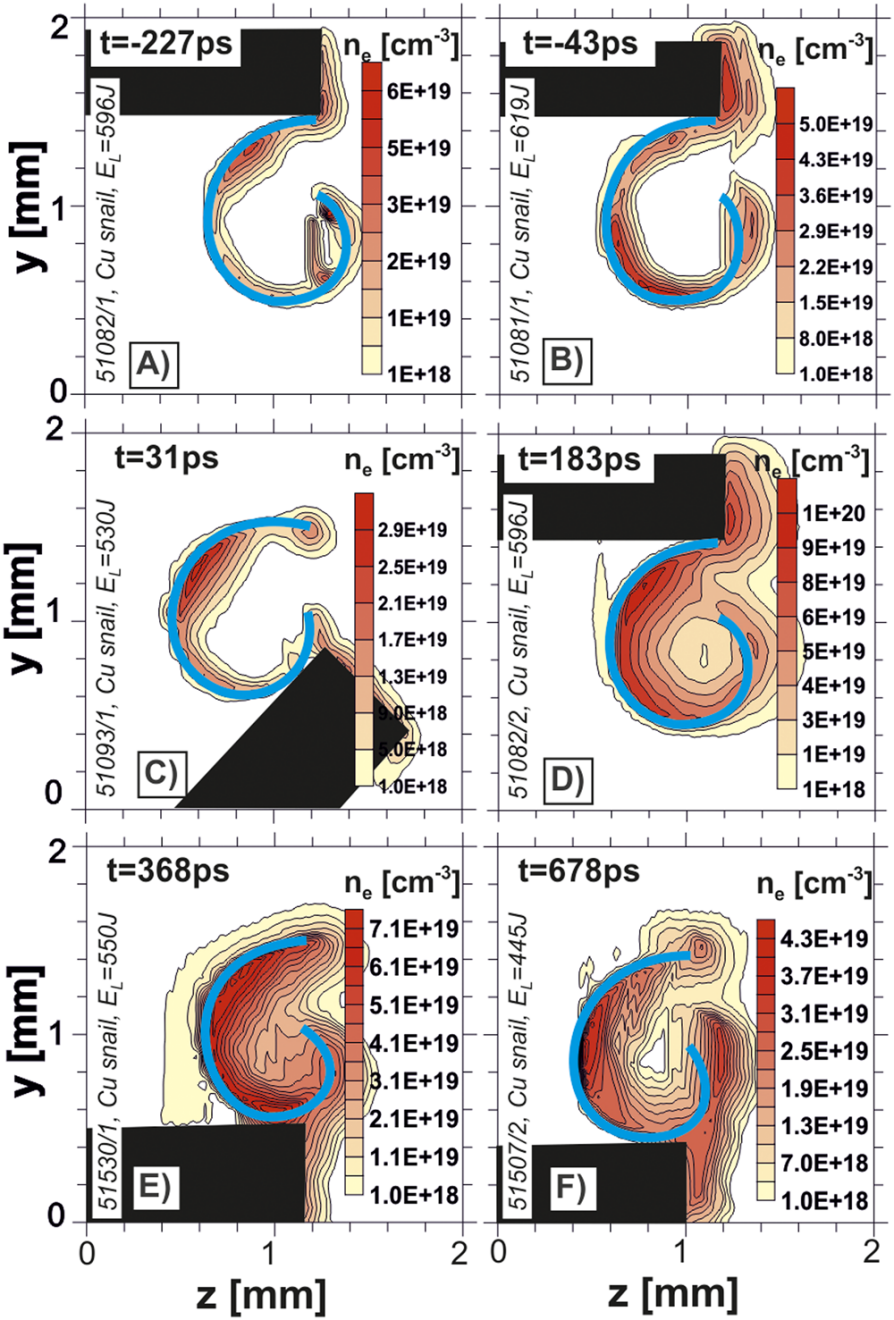
Electron density distributions in targets for the temporal sequence shown in Fig. 2.

Analysis of the raw interferograms, described in details in the Online Supplementary Information, allows extracting 2D distributions of the plasma density and the magnetic field. For the quantitative analysis, an important assumption was made concerning the plasmoid length along the target axis, *l*_*x*_ = 400 *μm*. This assumption was supported by the X-ray streak camera data. Reconstructed maps of the electron density distribution are presented in Fig. 3.

Already at the first time moment of −227 ps the plasma starts to expand inside the target, see Fig. 3A). When the main part of the driver pulse arrives, the expansion into the target volume continues. Starting from 183 ps, the plasma forms a regular structure inside the target hollow. The electron plasma density measured at the center of the snail target is about 10^18^ cm^−3^ and stays on this level at least for the next ~500 ps. Later, the increased plasma density did not allow to use the optical diagnostics.

**Figure 4:**
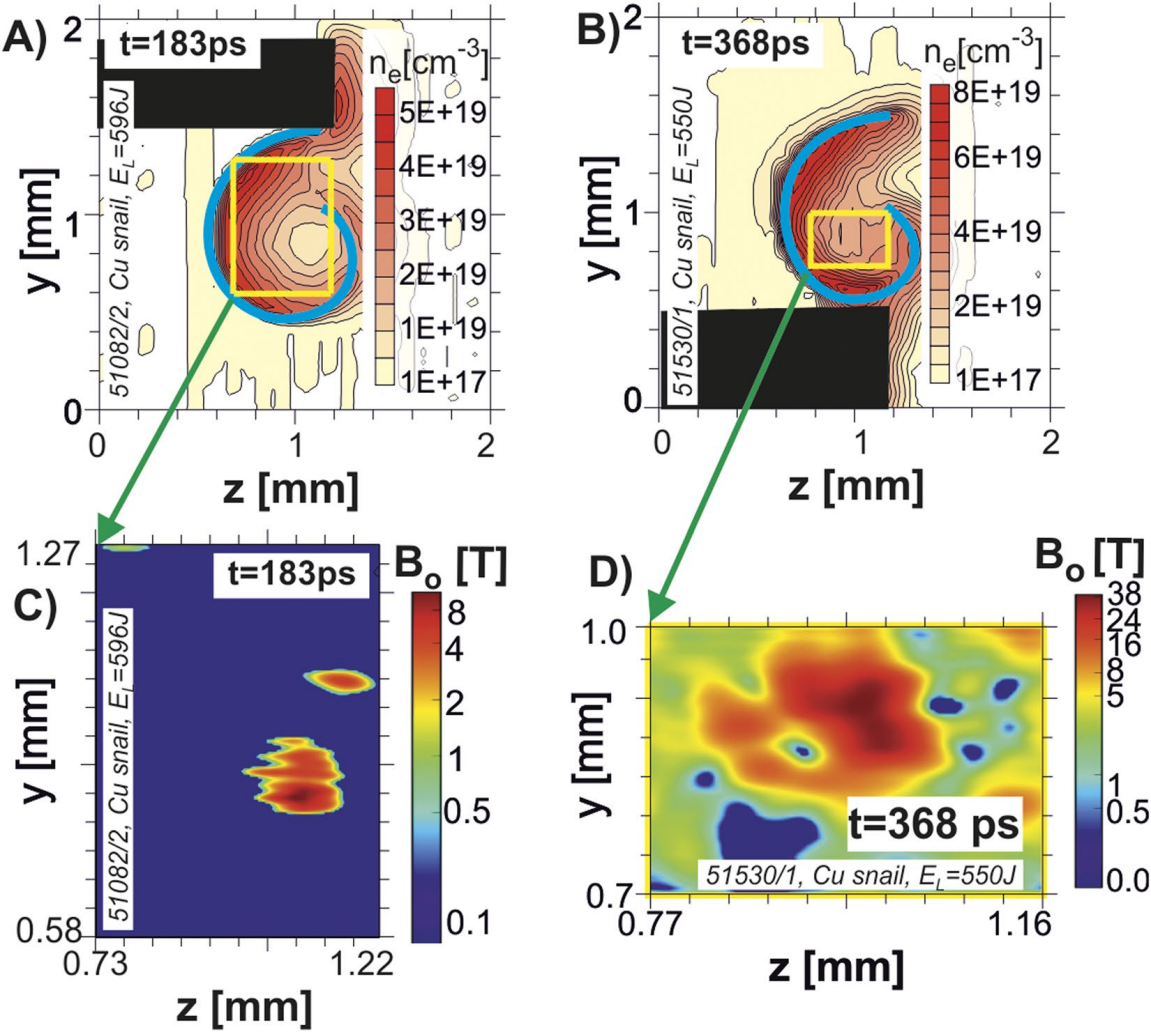
Density profiles (panels (A) and (B)) and magnetic field *B*_*x*_(*y*, *z*) reconstruction inside the target, based on polarimetry measurements at 183 ps (panel (C)) and 368 ps (panel (D)).

As shown below, this structure represents a theta-pinch configuration. The magnetic field in such structure is directed along the target symmetry axis, as visualized by the Faraday effect for the time moments 183 ps, 368 ps, and 678 ps. In Fig. 4 we present the qualitative data corresponding to 183 ps and 368 ps. Note that to estimate the magnetic field distribution *B*_*x*_(*y*, *z*), the density distribution ${\bar{n}}_{e}(y,\,z)$ obtained in the previous step of the data evaluation was involved. For details on the reconstruction algorithm and the methodology used we refer to the Methods section. In Fig. 4, the maximum amplitude of the magnetic field ranges from about ≈8 T at *t* = 183 ps to ≈40 T at *t* = 368 ps.

### Electron and ion emission

**Figure 5:**
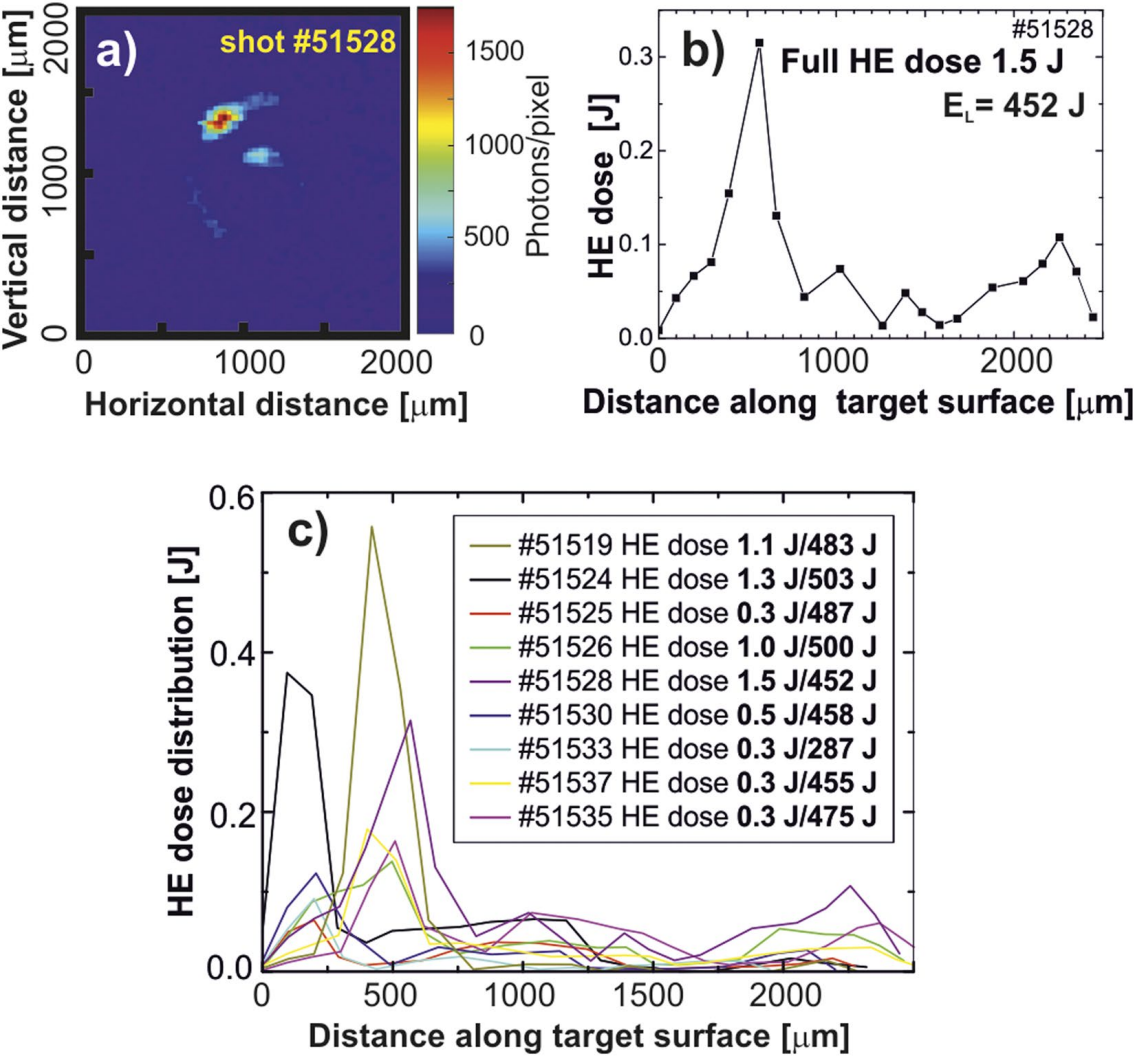
2D maps of the Cu K_α_ emission from the snail for the shot #51528 (panel a), the energy deposited by fast electrons along the internal surface of the snail in the shot #51528 (panel b), and the deposited energy variation in different shots (panel c).

The X-ray Kα imaging utilizing the second order reflection from the spherically bent crystal of quartz (211) provided 2D-resolved information on the production of fast electrons and the corresponding energy deposition in the targets bulk. The hot electron induced Cu Kα emission is conditioned by two factors: i) the presence of hot electrons with the energy above the K-edge ionization limit (i.e., above approximately 9 keV) and ii) their deposition in the relatively cold target material, otherwise the K-shell emission would be shifted to higher photon energies which are not covered by the imaging system used^19^. The details on experimental setup, reconstruction of the measured data and their evaluation in terms of the hot electron energy distribution along the target surface^12^ are provided in the Methods Section and Online Supplementary Information. An example of the measured Cu Kα signal is presented in Fig. 5a). This data indicates the energy deposition of fast electrons along the target surface and allows to estimate their total energy. The corresponding distribution of the fast electron dose along the target surface obtained by evaluating the Cu Kα signal is shown in Fig. 5b). According to Fig. 5(a) and (b), the maximum of the fast electron energy deposition coincides with the laser focal spot position. Statistical data on energy distributions for various shots are presented in Fig. 5c).

**Figure 6:**
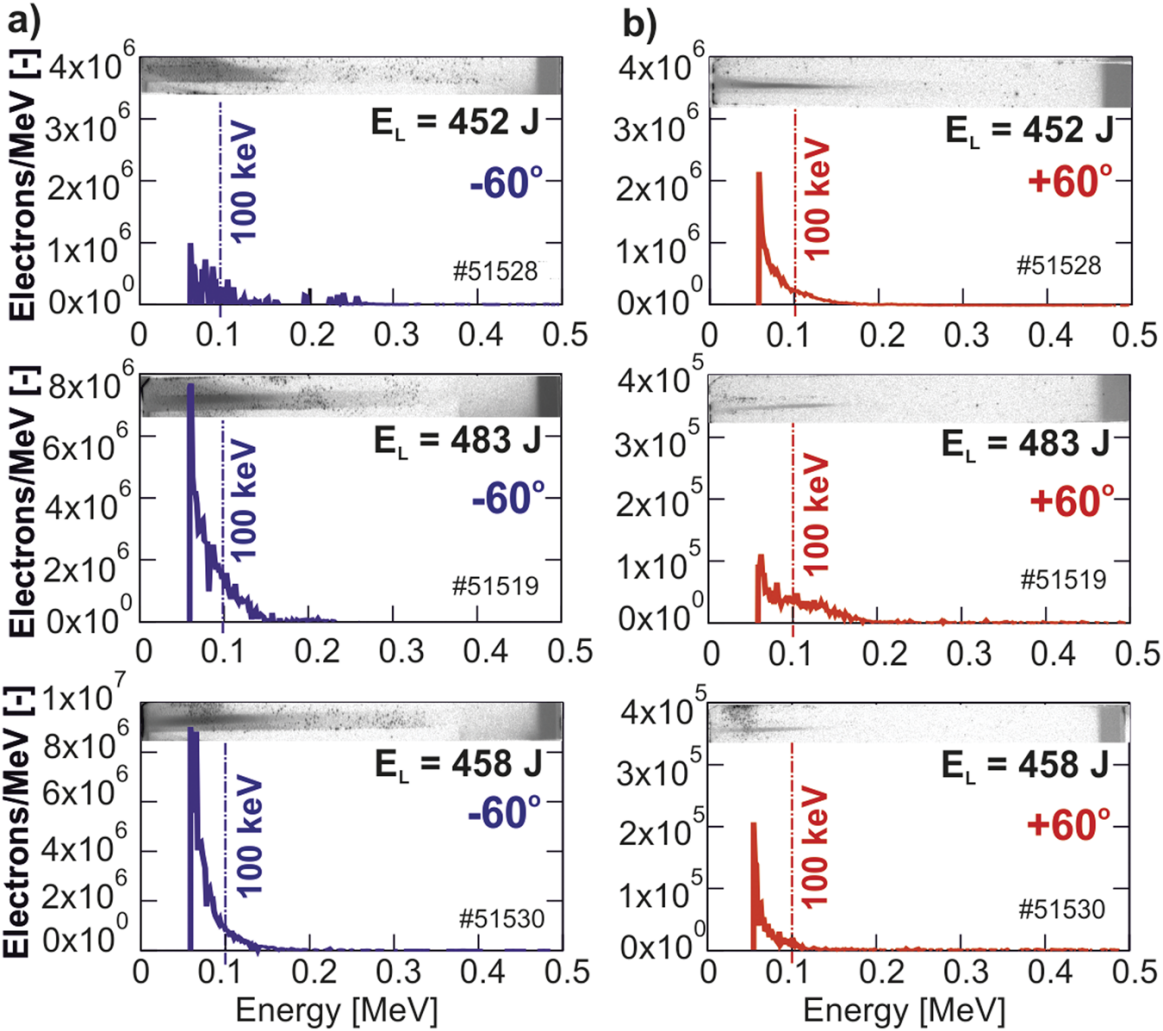
Electron spectra recorded in different shots using the magnetic electron spectrometer and observed in two directions: (**a**) −60° and (**b**) +60° in relation to the target axis.

Data obtained from electron spectrometers for several shots are shown in Fig. 6. The energy distributions of electrons emitted in the symmetrical directions are very similar, the maximum electron population corresponds to the energy of tens keV, see Fig. 6. The difference in electron emission observed in several shots probably results from an asymmetry caused by the not fully reproducible alignment and shape of the targets.

**Figure 7:**
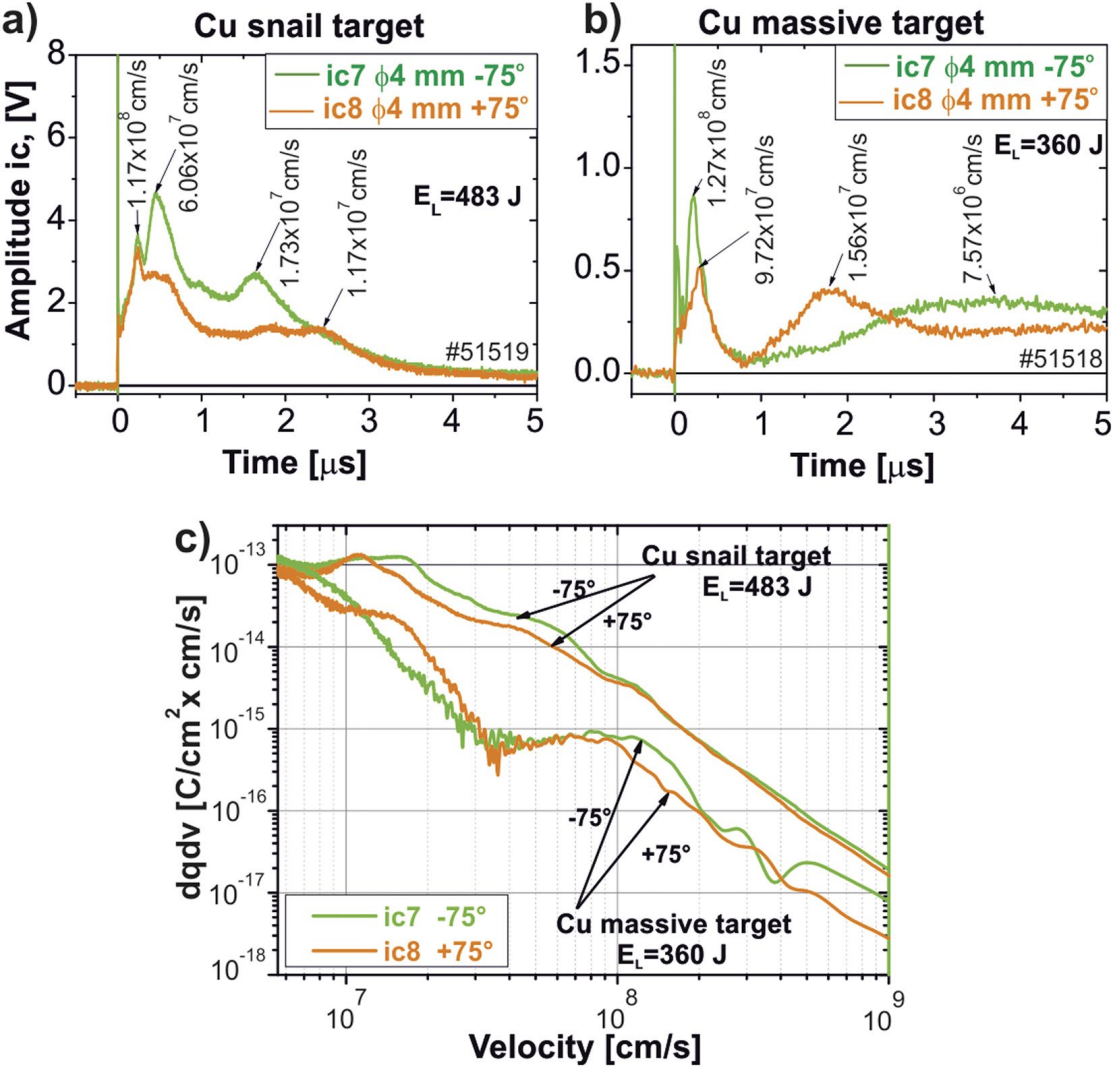
The ion collector signals registered at two opposite directions, −75° and + 75° to the target axis, for the snail target (panel a)) and Cu massive flat target (panel b). The ion charge density –velocity distributions for the snail and the Cu massive targets (panel c).

Ion collector data is shown in Fig. 7a). For comparison, the ion collector data measured using the Cu massive flat target at the same laser beam parameters is shown in Fig. 7b). Details concerning the experimental conditions for investigation of the flat massive targets are presented in the Online Supplementary Information. The signal is about an order of magnitude stronger compared to the snail targets. This indicates qualitative changes in the plasma expansion process.

Such qualitative difference can be seen even more clearly when comparing the charge density distribution in the velocity domain for Cu snail and massive targets, cf. Fig. 7c). For both ion collectors, *dqdv* for the velocity range from 10^7^ to 10^9^ cm/s is significantly higher for the snail than for the massive targets. Ion velocity distribution from the snail targets is a linear function for a broad velocity range, whereas it has an irregular shape for massive targets.

### Return current

**Figure 8:**
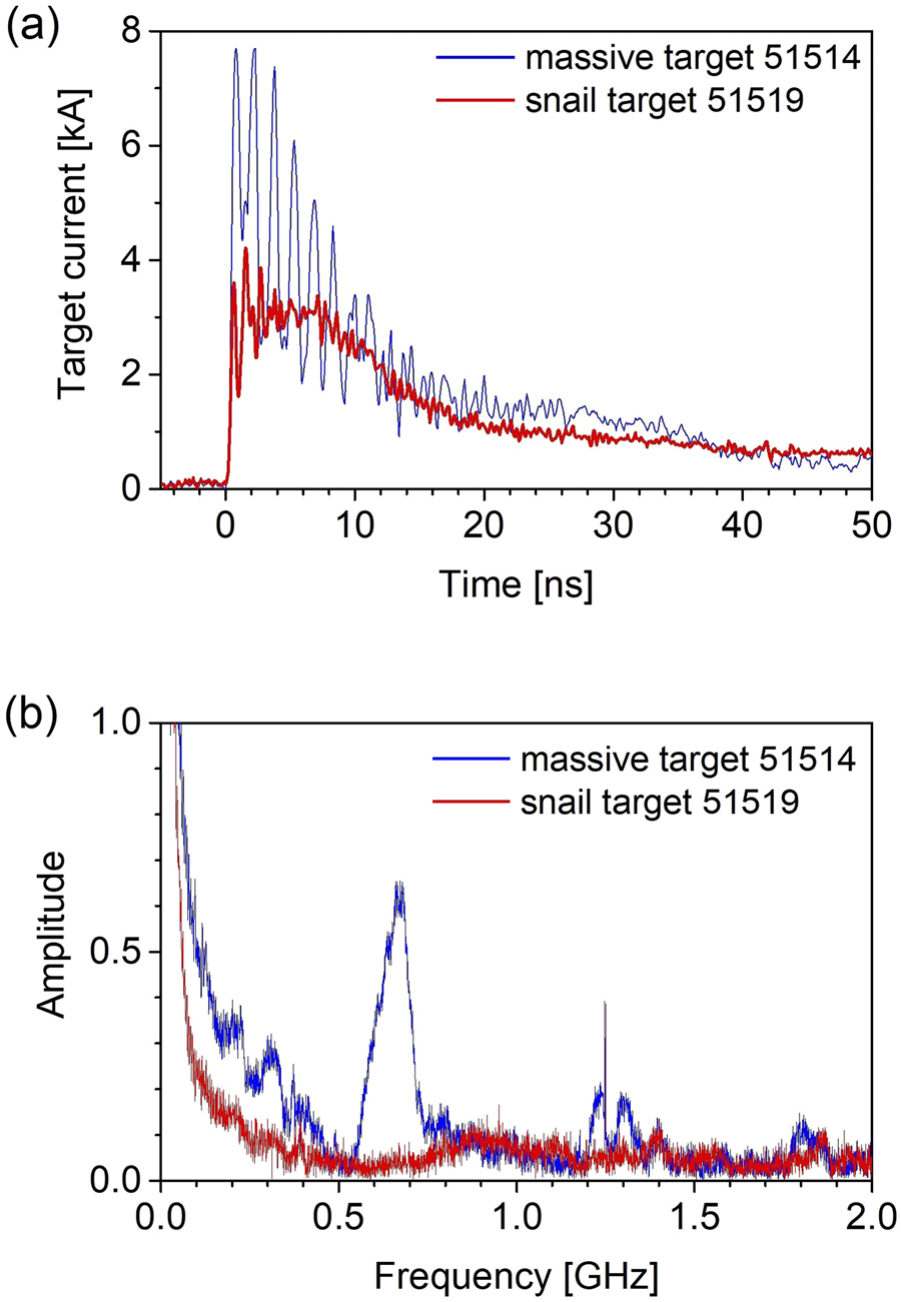
Comparison of return currents signal (panel (a)) and their frequency spectra (panel (b)) for the Cu massive flat target and the snail-shaped target.

An important parameter characterizing the laser-target interaction is the total return current, which relates to the electrons escaping completely the target^15,20^. Figure 8 shows the return currents *J*_*MT*_(*t*) and *J*_*ST*_(*t*) from the massive flat (MT) and snail targets (ST), respectively, and their frequency spectra. The peak amplitude of *J*_*MT*_(*t*) is higher by a factor of ∼2 than the peak of *J*_*ST*_(*t*). The ∼100-ns duration of both target currents is much longer than the duration of the laser-target interaction, similar scale was observed in other experiments^21,22^. It is obvious that the *J*_*MT*_(*t*) is strongly modulated by a frequency of about 670 MHz which matches the resonant frequency of the target holder with the length of 11 cm. Figure 8 clearly shows the influence of the snail target shape on the mitigation of resonant frequencies of the target and the target holder system. Moreover, the long-time return current pulse is more extended for the snail targets which supports the observation of a localized plasmoid in other diagnostics, see the frequency spectra over the range of 10–500 MHz shown in Fig. 8b). The time-integral of the target current allows to estimate the number of electrons escaping the target. For the massive target we found about 5 × 10^14^ and about 3 × 10^14^ electrons for the snail target in the time interval of 50 ns.

### X-ray emission

**Figure 9:**
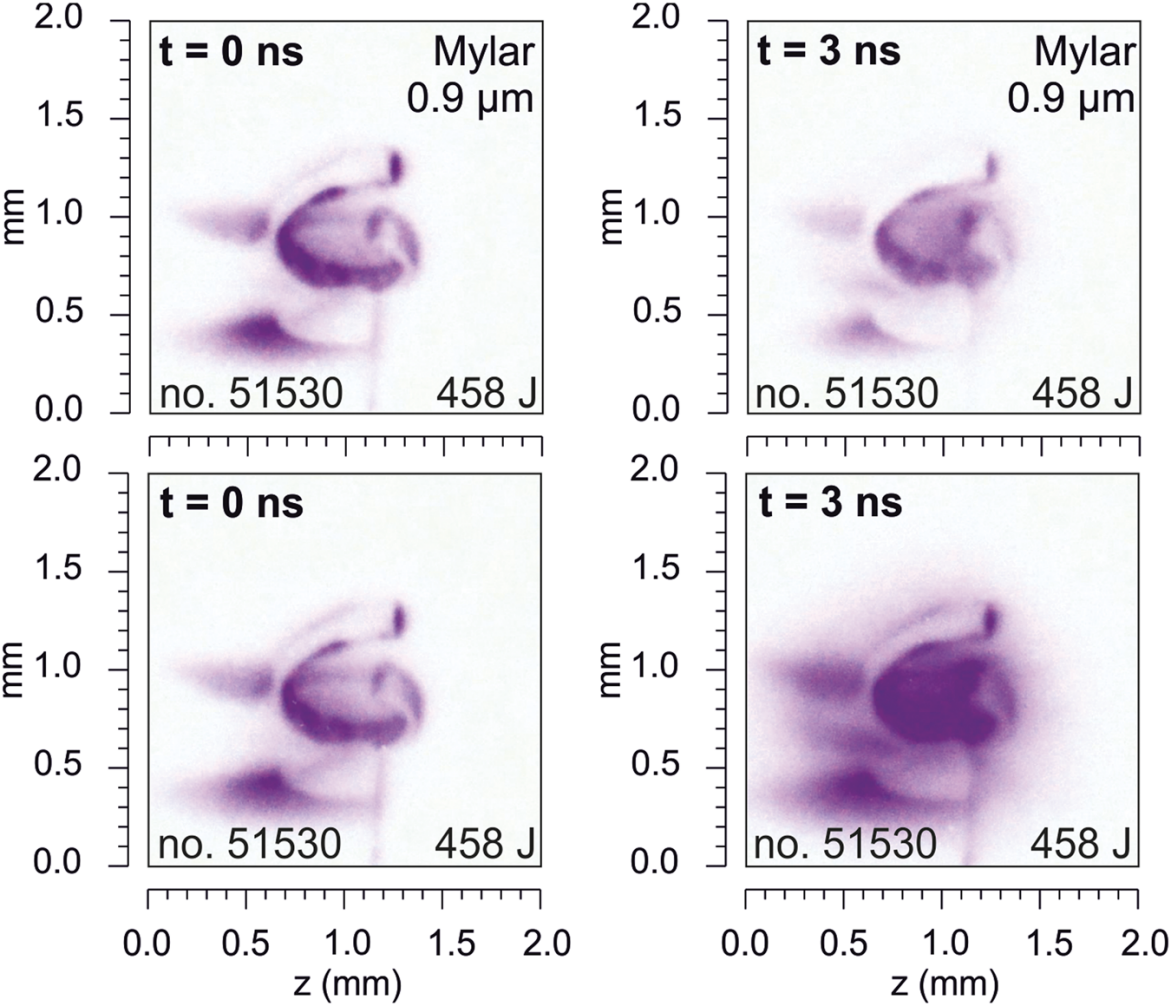
Gated soft x-ray images registered by the 4-frame pinhole camera which shows snail-shaped target self-emission at 0 and 3 ns with and without MYLAR filter.

The soft x-ray emission data is presented in Fig. 9. Four-frame soft x-ray pinhole camera visualizes the plasma formation process in snail-shaped targets. The soft x-ray emission is observed both along the target surface and from the magnetized plasma inside the target volume. Note that because of the orientation of the X-ray camera, the region of the primary interaction of the laser beam with the target is hidden behind the upper part of the snail. In several shots, we filtered soft x-ray radiation with a 0.9 µm Mylar filter. At the time of laser interaction, i.e., at about t = 0 ns, there is not a significant difference between images taken with and without 0.9 μm Mylar filter. This means that for this time, most of the photons have energies above 100 eV. The self-emission of the hot plasma inside the snail is clearly visible later in time when the plasma implodes to the center of the target and the optical diagnostics fails. As Fig. 9 shows, the self-emission at t = 3 ns seems to be even more intense. However, since a significant part of the plasma self-emission is absorbed by a 0.9 μm thick MYLAR filter, a large number of photons are less energetic. Further details can be found in the Online Supplementary Information.

## Discussion

The grazing incidence of the laser onto the curved surface results in a continuous light reflection along the internal target wall. This effect, observed earlier for a relativistic pulse^10^, is confirmed by the data shown in Fig. 9. The whole internal surface of the target almost uniformly emits soft X-rays. In Fig. 5, the energy deposition by the hot electrons shows a peak near the entrance of the laser beam, for different shots from 100 to 500 μm, and a very slow decay along the rest of the target, up to its opposite side. Such a long interaction length leads to a very efficient absorption and the laser energy deposition in the target. At the same time, the emission of the hot electrons is suppressed due to the decrease of the beam intensity at a large incidence angle and due to a presence of the magnetic field in the target interior. The Larmor radius for the hottest part of the electron distribution (~100 keV, see Fig. 6) in the magnetic field of ~50 T is about 20 μm, which is much less than the target size. This suppression is indicated by the reduced value of the total discharge current in the snail target in comparison to the flat massive target, cf. Fig. 8.

However, the local discharge currents in the snail target are of a great importance. Within the grazing incidence geometry, the azimuthal symmetry of the plasma expansion and hot electron generation inherent for flat normally irradiated targets^5^, breaks, and both kinetic and thermo-currents are no more efficient for the magnetic field generation. In contrast, the discharge currents governed by the target geometry appear to represent the effective magnetic field source.

Besides the irradiation geometry, the laser intensity in the considered situation of the moderate-intensity pulse is not sufficient for a direct electron acceleration, as it was in the relativistic case^6^. In this intensity domain, the efficiency of the magnetic field generation is known to be less^8^, since the discharge currents almost solely contribute to the local magnetic field in the ablation region. In the relativistic case, the second strong current, which closes the local current loop at the initial stage of the interaction process, is the current of accelerated electrons along the surface^6^. Due to these reasons, the magnetic field may be estimated by the efficiency of laser energy transfer to fast electrons.

The Cu Kα measurements indicate that 0.1–0.3% of the laser energy, i.e., of the order of 1 Joule, refers to the hot electrons which eject K-shell electrons from the relatively cold material of the target and result in bound-bound atomic transitions. There is also a certain amount of the hot electrons which do not interact with the target bulk, remain in the hot ablating plasma or escape from the interaction region into the vacuum. These electrons are not seen by Cu Kα measurements but contain a part of the laser energy, thus the fraction of the total laser energy transformed to the hot electrons should be corrected. The simplest assumption of the symmetric distribution results in a multiplication by a factor of 2, so that the total hot electron energy is 0.2–0.6%. Another correction comes from the target geometry. For hot electrons with energy 30–100 keV the stopping power in a solid copper is 2–6 MeV cm^2^/g^23^. This results in the range of 10–100 μm. The target thickness is 20 μm, consequently the more energetic part of electrons can create Kα photons in the initial part of their trajectory only when passing the target bulk. The fraction of hot electrons escaping to vacuum is not seen by the Cu Kα measurements which may result in 3–5 higher value. Taking into account these two corrections, the energy contained in hot electrons may reach few percent, though a more accurate estimate is problematic.

For the estimate below consider the absorbed energy as 2–4% in the hot electrons with the average energy ~100 keV. Though these electrons may remain in the interaction region, as follows from the small total discharge current, locally they act as a driver for discharge currents in the ablating plasma. This corresponds to 2–5 × 10^14^ electrons in ~400 ps. Then the current is 200–400 kA, the value about few times more than that expected for capacitor-coil targets for similar irradiation conditions^2^. This current at the characteristic length of *d* ~ 100 μm produces magnetic field *B*_0_ ~ 800*T*, which magnetizes dense surface plasma of the order of critical density *n*_*e*_ ~ 10^21^ cm^−3^. Plasma ablation and expansion to the internal volume leads to the density drop down to *n*_*e*_ ~ 10^19^ cm^−3^, and the frozen magnetic field drops as $B\approx \frac{{B}_{0}{n}_{e}}{{n}_{s}}\approx {10}^{-2}{B}_{0}\, \sim \,8\,{\rm{T}}$, to conserve the magnetic field flux. This estimate appears to be consistent with the measured data presented in Fig. 4(C).

According to Fig. 3, the plasma edge expansion velocity is about 2·10^8^ cm/s, in accordance with the standard energy relations. Considering the heated surface square to be ~2πR_t_·2R_L_, where R_t_ is the target radius and R_L_ is the focal spot radius, the plasma temperature T may be related to the effective temperature T_eff_ for the normal incidence as T ≈ T_eff_ R_L_/4R_t_. Assuming isothermal expansion, the effective temperature is defined as T_eff_ ≈ C_V_^−1^(I/ρ_cr_)^2/3 ^^24^, where С_v_ = (Z + 1)k_B_/(γ + 1)Am_p_, Z and A are the average ion charge and the ion number, m_p_ is the proton mass, γ is the adiabatic index, k_B_ is the Boltzmann constant, ρ_cr_ (g·cm^−3^) = 1.8·10^−3^ A/Zλ^6^ is the mass critical density, and λ is given in μm. Then the sound velocity is c_s_ = (C_V_T)^1/2^ ≈(R_L_/4R_t_)^1/2^(I/ρ_cr_)^1/3^. Considering the expanding Cu plasma for I = 2 × 10^16^ W/cm^2^, Z = 19, ρ_cr_ = 3.1·10^−3^ cm^−3^, R_L_ = 50 μm, and R_t_ = 500 μm, we obtain c_s_ ≈ 6.3·10^7^ cm/s. The low-density plasma edge expands with the velocity 2с_s_/(γ − 1), which is 3с_s_ for γ = 5/3, consequentlythe implosion velocity may be estimated as 1.9·10^8^ cm/s, thus supporting the observed values.

It may be expected that in the process of magnetized plasmoid formation, the amplitude of the frozen magnetic field would increase as a result of the radial implosion^6^ by hot dense plasma coming from the target walls. Within the actual experiment, this expectation was confirmed up to the time of a few hundreds ps after the laser pulse termination. Later on, reliable polaro-interferometric measurements of the magnetic fields become more difficult or even impossible due to illegibility of interferograms. In Fig. 4, the two stages of the plasma implosion are seen. Just after the laser pulse end (183 ps), the magnetic field scale is about 5–10 T. Later, at 368 ps, the plasma expands in the interior of the target, approaching an equilibrium $\,{n}_{e}{T}_{e}\, \sim \,\frac{{B}^{2}}{8{\rm{\pi }}\,}$. For conditions of our experiment, the relevant plasma temperature may be estimated as 1 keV^5^ and the density is measured to be *n*_*e*_ ~ 10^19^ cm^−3^, see Fig. 4B). This gives the scale of the equilibrating magnetic field in the central part *B* ~ 40 – 60T which corresponds to the measured value in Fig. 4(D). From this estimate we can see that the theta-pinch equilibrium is approaching at this stage, when the thermal and directional components of the plasma energy are of the same scale, and the field compression starts to saturate. To achieve higher degree of compression, more laser energy deposition per plasma unit volume is necessary, either by increasing the laser pulse energy or by decreasing the target size.

As shown above, by application of appropriate geometry of the laser-target irradiation in the moderate-intensity TW regime, it is possible to create a stable, long-living magnetized plasma structure. The magnetic field strength in the reported experimental study was about several tens of Tesla. Although this value may be obtained by other techniques, in the considered situation it appears to be frozen in the hot laser plasma^6^. In other words, this method allows to produce magnetized plasma with a controlled magnetization which may be advantageous for more complicated astrophysical experimental studies like the collisionless or low-collisional magnetized shock wave generation^25^, particle acceleration^26^, jet formation^27^, reconnection^28^, and many other phenomena.

## Methods

In this experimental study, the main diagnostic tool was the complex interferometry^18^. It was used to obtain information about both the electron density distributions and magnetic fields in the plasma formed inside of a snail target in 2-frame regime. Within this approach, the full set of data related to an optical diagnostic of a single laser shot was obtained in two consecutive time moments by the phase - amplitude analysis of the interferometric fringes. The polaro-interferometer was driven by a Ti:Sa laser pulse (808 nm and 40 fs) synchronized with the iodine PALS laser^17^.

First, the average electron density distribution ${\bar{n}}_{e}(y,\,z)$ was calculated from the fringe deviation. Because of a strong disturbance of the Fourier spectrum by the snail target and holder construction, the method of maximum fringes was applied for the phase distribution reconstruction of interferometric patterns in complex interferograms, see details in the Supplementary Information.

For a quantitative analysis, the plasma length *l*_*x*_ = 400 *μm* derived from the X-ray streak camera measurements was assumed. Figure 3 shows an average electron density distribution of plasma structures formed in the snail targets at several time moments, the corresponding raw images are presented in Fig. 2. The magnetic field amplitude in these structures was estimated via Faraday effect of the polarization plane rotation of the linearly polarized auxiliary laser beam^29^. The distribution of the Faraday rotation angle was based on the amplitude analysis of the Fourier spectrum of complex interferograms. The results of this procedure applied to two complex interferograms (corresponding to Fig. 2(D) and (E)) are shown in Fig. 4. To eliminate the effect of the target and holder disturbance on the Fourier spectrum, only marked areas (yellow rectangles in Fig. 4(A) and (B)) inside the targets were analyzed.

For acquisition of 2D-resolved images of the Cu Kα emission from laser-irradiated snail targets cf. Figure 5, a spherically bent quartz crystal was used. This combination of the radiation with the photon energy of 8047.8 eV and the refractive-index corrected crystal interplanar spacing 2d = 0.15414 nm results in the quasi-normal incidence configuration of the imaging system with a negligible distortion due to diffraction from sagittal and meridional planes of the crystal. The system provided the magnification of 1.73, the distortion of images due to an inclined target observation was taken into account within the reconstruction procedure. The transfer function of the system was calculated using the ray-tracing algorithm following theoretical approach formulated by Podorov *et al*.^30^. Recorded signals were recalculated to an intensity scale taking into account the crystal reflectivity and transmission through protective filters. A Monte Carlo code PENELOPE^31^ was used to model the hot electron energy deposition into the target material and subsequent production of Kα emission. Finally, the measured x-ray data were interpreted in terms of hot electron generation and conversion efficiency of the laser radiation energy into hot electrons, respectively. Details of this methodology are presented in the paper^12^, see also the Supplementary Information^32–34^.

The electron spectrometers were positioned in the horizontal plane symmetrically around the target. The spectrometers used permanent magnets with the field strength of 0.2 T, the signal was detected by BAS-SR imaging plates. From the scanned images, the electron spectrum and background data were obtained and further processed to obtain the real numbers of electrons impinging on the image plate, see details in the Supplementary Information. Figure 6 presents selected electron spectra obtained in several shots with similar laser energy.

Ion measurements were performed by ToF (Time of Flight) technique. The data were collected with several Faraday cups situated at an angle of ±75° with respect to the target normal. The detected signal was independent on the ion energy, consequently the signal amplitude is proportional to the ion flux current. Sample results of these measurements are shown in Fig. 7

The results of the target current measurement are shown in Fig. 8. The target return current probe was mounted between a metal target holder and a manipulator with conductive connection to the vacuum chamber. Technical details are presented in the Supplementary Information.

A four-frame X-ray pinhole camera with approximately ns exposure and 3 ns interframe separation was used to measure extreme ultraviolet and soft x-ray emission of the laser-produced plasmas, cf. Fig. 9 for sample results and the Supplementary Information for technical details.

## Electronic supplementary material

Supplementary information
