# Supplementary material for: Magnetized plasma implosion in a snail target driven by a moderate-intensity laser pulse
Source: Sci Rep. 2018 Dec 17;8:17895. doi: 10.1038/s41598-018-36176-8 (PMC6297252; doi:10.1038/s41598-018-36176-8)
Supplement: Supplementary file 1 — Supplementary information [file 41598_2018_36176_MOESM1_ESM.pdf]

Magnetized plasma implosion in a snail target driven by a moderate-intensity laser pulse

T. Pisarczyk[1], S. Yu. Gus'kov[3,2], A. Zaras-Szydłowska[1], R. Dudzak[4,5], O. Renner[4,5], T. Chodukowski[1], J. Dostal[5,4], Z. Rusiniak[1], T. Burian[4,5], N. Borisenko[2], M. Rosinski[1], M. Krupka[5], P. Parys[1], D. Klir[6,5], J. Cikhardt[6,5], K. Rezac[6,5], J. Krasa[5], Y.-J. Rhee[7], P. Kubes[6], S. Singh[4], S. Borodziuk[1], M. Krus[5,4], L. Juha[4,5], K. Jungwirth[4], J. Hrebicek[4,5], T. Medrik[4,5], J. Golasowski[4,5], M. Pfeifer[4,5], J. Skala[5,4], P. Pisarczyk[8] and Ph. Korneev[2,3]

[1] Institute of Plasma Physics and Laser Microfusion, Warsaw, Poland
[2] P.N. Lebedev Physical Institute of RAS, Moscow, Russian Federation
[3]National Research Nuclear University MEPhI, Russian Federation
[4]Institute of Physics, Czech Academy of Sciences, 182 21 Prague, Czech Republic
[5]Insitute of Plasma Physics, Czech Academy of Sciences, 182 00 Prague, Czech Republic
[6]Faculty of Electrical Engineering, Czech Technical University, 166 27 Prague, Czech Republic
[7]Center for Relativistic Laser Science, IBS, Gwang-Ju, 61005, Korea
[8]Warsaw University of Technology, ICS, Warsaw, Poland

ONLINE SUPPLEMENTARY INFORMATION

Complex interferometry

To obtain information about the electron density and magnetic fields distribution in the plasma formed inside the snail target, 2-frame polaro-interferometer operating in the complex interferometry regime was applied[18], see Fig. S1. Within this approach, the sought information can be obtained directly from one image based on the phase-amplitude analysis of interferometric fringes. The polaro-interferometer was using a Ti:Sa laser pulse (808 nm and 40 fs) synchronized with the iodine PALS laser as described in paper[17].

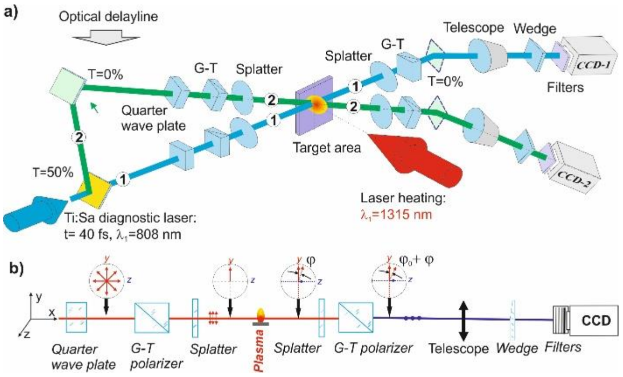

Fig. S1: 2-frame polaro-interferometric system: a) general view and b) optical scheme of one-frame module for registration of the complex interferograms.

According to the geometry of the optical probing, distribution of the electron density and magnetic field in the (x,y) plane normal to the probing direction has been calculated from average phase, the Faraday rotation data, and assuming a given plasma probing length along the x-direction.

The average electron density distribution, $\bar{n}_e(y,z)$ can be calculated using the relationship between the phase and electron density in a fully ionized plasma[32]

$$\delta(y,z) = 4.46 \cdot 10^{-14} \lambda \bar{n}_e(y,z) l_x \qquad (1)$$

where δ (y, z) increases the phase of the probing beam with the wavelength λ along the plasma with the length l_x. Average electron density distribution is defined as

$$\bar{n}_e(y,z) = \frac{\int_0^{l_p} n_e(x,y,z)dx}{l_p} \qquad (2)$$

However, it was not possible to apply the phase analysis of the complex interferograms[18] due to a strong disturbance of the Fourier spectrum by the snail target and holder construction. Therefore the phase distribution $\delta(y,z)$ was determined by reconstruction of interferometric fringes on complex interferograms using the *method of maximum fringes*[33].

Calculations were made for the length l_x=400 μm, an average value of which was estimated from the X-ray streak camera measurements. Average electron density distributions of plasma streams formed in the snail targets obtained by evaluation of interferograms from Fig. 2 are depicted in Fig. 3 of the main text. These distributions clearly envisage formation of the plasma configuration with axial symmetry and the electron density at the center of the snail target about 10^{18} cm^{-3}. The plasma is magnetized with the magnetic field line direction along the snail axis. This conclusion is supported by the Faraday effect visible on the complex interferograms shown in Fig, 2 of the main text for the time moments 183 ps and 368 ps. To estimate the magnetic field amplitude in the magnetized plasma, the formula for the Faraday angle rotation of the linearly polarized laser beam was used[29]

$$\varphi(y,z) = 2.62 \times 10^{-17} \lambda^2 \bar{n}_e(y,z) \bar{B}_o(y,z) l_x\,, \qquad (3)$$

where: $\bar{n}_e(y,z)$ and $\bar{B}_o(y,z)$ are distributions of the average electron density and the magnetic field, respectively, and l_x is the length of the plasma.

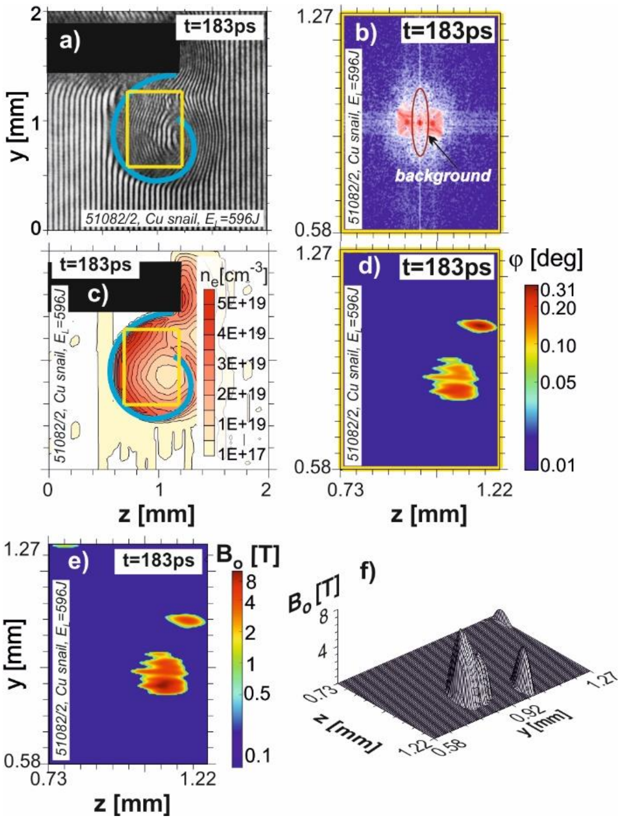

Fig. S2. The complex interferogram obtained for t= 183 ps (panel a)), its Fourier spectrum (panel b)), electron density (panel c)), rotation angle (panel d)) and the magnetic field distributions (panels e), f)).

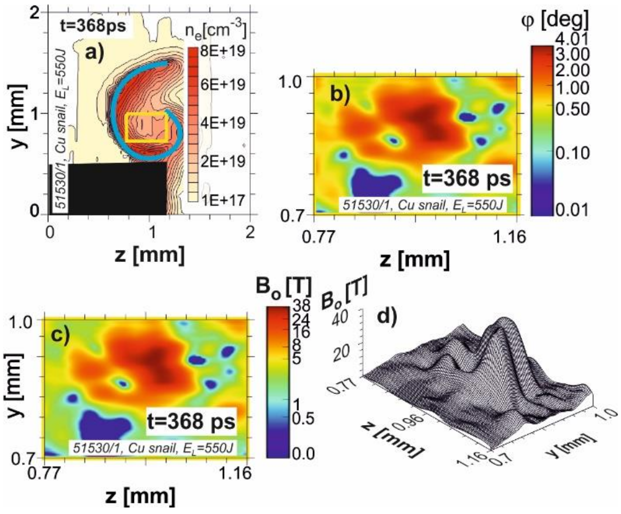

Fig. S3 The results of the quantitative analysis of the complex interferogram recorded at t= 368 ps: a) electron density, b) rotation angle, c), d) the magnetic field distributions.

The distributions of the Faraday rotation angle were determined based on the amplitude analysis of the Fourier spectrum of complex interferograms. Results of the quantitative analysis of selected complex interferograms (corresponding to Fig. 2 in the main text) are shown in Figs S2 and S3.

According to the methodology described in the paper[18], the central part of Fourier spectrum (the so-called background) corresponds to the intensity distribution of interferometric fringes connected with the Faraday rotation angle, as shown in Fig. S2b. To eliminate an influence of the target and holder construction on Fourier spectrum, only marked areas (yellow rectangles) inside snail targets were analyzed.

Cu-Kα measurements

The 2D-resolved images of the Cu Kα emission from laser-irradiated snail targets were obtained using a quartz (422) crystal, spherically bent to a radius of 380 mm. This combination of the radiation with the photon energy of 8047.8 eV and the refractive-index corrected crystal interplanar spacing 2d = 0.15414 nm results in the quasi-normal incidence configuration of the imaging system with a negligible distortion due to diffraction from sagittal and meridional planes of the crystal. The images were recorded onto the FUJI BAS MS imaging plates and scanned using the Fujifilm BAS-1800 reader with a pixel size of 50×50 μm^2. The system provided the magnification of 1.73, the distortion of images due to an inclined target observation (23.5° from the x-axis of the snail-shaped targets) was taken into account within the reconstruction procedure. The transfer function of the system was calculated using the ray-tracing algorithm following theoretical approach formulated by Podorov et al[30]. Recorded signals were recalculated to an intensity scale taking into account the bent crystal reflectivity and transmission through protective filters. A Monte Carlo code PENELOPE[31] was used to model the HE energy deposition into the target material and subsequent production of Kα emission. Finally, the measured x-ray data were interpreted in terms of the HE generation and conversion efficiency of the laser radiation energy into hot electrons, respectively. Details of this methodology are presented in the paper[12].

Electron spectrometer measurements

Two electron spectrometers were placed at 60 degrees on both sides of the target in the horizontal plane. The electrons escaping the plasma in the target area follow their path into the entrance aperture of the spectrometers, where they are deflected in the magnetic field and detected by the BAS-SR imaging plates.

The recorded spectra were afterward scanned using the Fujifilm BAS-1800 II imaging plate reader. From the scanned image of the electron spectrum, signal and background data traces were obtained and further processed to obtain the real numbers of incident electrons on the image plate, see Fig. S4.

The calibration curve relating signal position on the imaging plate to the energy of incident electrons was determined via mapping the magnetic field inside the spectrometer gap with a numerical tracking of the electrons performed by the SIMION software[34].

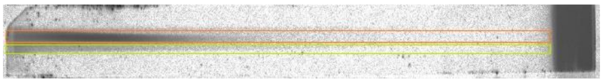

Fig. S4: Imaging plate scan with signal *(orange) and* background *(green) data traces.*

Electron energy is given by formula below where energy E is in keV and distance x is in mm:

$$E(x) = (0.4998x^2 - 7.1465x + 80.034). \qquad (4)$$

The scanner output is digitized data in the 16-bit depth, also called quantum level (QL). Those were converted to PSL level values (S_{PSL}) using the formula

$$S_{PSL} = \left(\frac{R}{100}\right)^2 \left(\frac{4000}{S}\right) 10^{L\left(\frac{QL}{G}-\frac{1}{2}\right)} \qquad (5)$$

where resolution R=50 µm, sensitivity S=4000, latitude L=5, and gradation G=65535.
Using the previously published calibration curve[13] shown in Fig. S5, the PSL level values were subsequently converted to electron numbers that were detected at a given resolution on the imaging plate. The calibration curve can be approximated by a Gaussian curve. This approximating function was further used to obtain the electron numbers on every sampled point of the electron spectrum. The characteristic values of the Gaussian function

$$f(x) = y_0 + A\exp\left(-\frac{(x-x_c)^2}{2w^2}\right) \qquad (6)$$

were absolute offset y_0=0.010, amplitude A=0.018, mean x_c=100 and width w=257.
The electron spectra obtained in several selected shots are shown in Fig.6 of the main text.

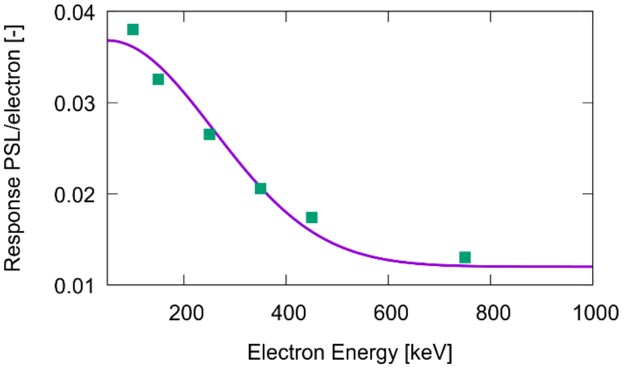

Fig. S5 Calibration curve of the BAS-SR imaging plate overtaken from[13].

Ion measurements

Ion measurements were realized by using the ToF (Time of Flight) technique. Data were recorded by several Faraday cups situated at angles ±75° with respect to the target normal in the horizontal plane. The recorded signals show different groups of ions emitted from the interaction region of target. The detector was fully independent on the ion energy, thus an amplitude of the ion collector signal is proportional to the ion flux current. The ion measurements provided information mostly on moderate energy particles but also on faster ions which are hard to detect by the ion

collector because of the short distance between the target and detector.

Target current measurements

Target current measurement system is schematically drawn in Fig. S6. The target current probe is mounted between a target holder and a target manipulator which is conductively connected to the vacuum chamber. Both the holder and manipulator are made of metal. Therefore a charge of the target is compensated by a return current through the holder, probe, and the manipulator to the grounded vacuum chamber. The current probe is based on the resistive shunt composed of 192 miniature (SMD 1206) 10-Ω resistors connected in parallel. Thus the total resistance of the shunt is 0.052 Ω. Using RG214 double shielded coaxial cable, the current probe is connected with the matched 3-GHz oscilloscope placed in the Faraday cage.

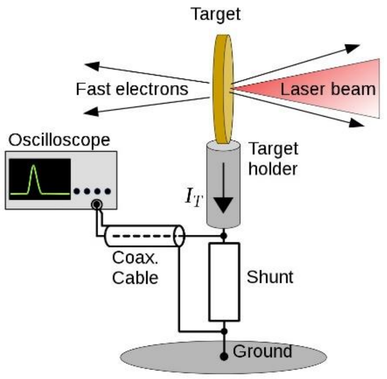

Fig. S6 Scheme of the taget current measurement.

Four-frame x-ray pinhole camera

A four-frame pinhole camera with approximately ns exposure and 3 ns interframe separation was used to measure the extreme ultraviolet and soft x-ray emission of the laser-produced plasmas[16]. As shown in Fig. 1 of the main text, the line of sight was normal to the laser axis. The plasma self-emission was transferred with the magnification of ~4 onto the microchannel plate (MCP) detector coupled with the digital camera Nikon D600 (6016 x 4016 pixels).

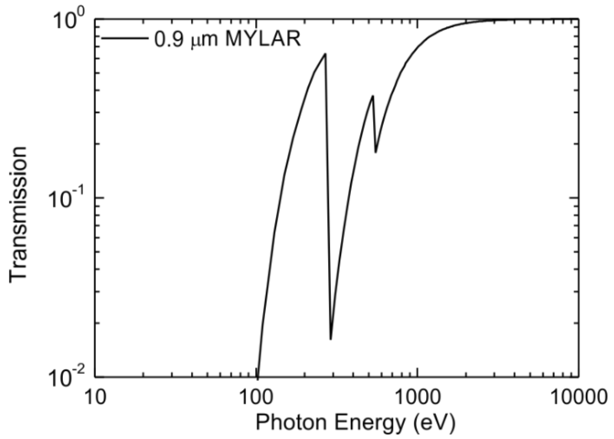

Fig. S7: Photon-energy dependence of the transmission of 0.9-μm-thick MYLAR filter.

This experimental geometry including the pinhole diameter of 35 μm resulted in the spatial resolution of about 45 μm. The MCP detector is sensitive to photons in the 10-10000 eV energy range. When using 0.9-μm-thick MYLAR filter, the photons with energy above 100 eV were detected (see Fig. S7 for transmission of the MYLAR filter).

Flat massive target experiment

The experimental setup for the flat targets was similar to that used for the snail-type targets. The massive Cu flat targets (cuboid shape with dimensions of 5x5x50 mm) were irradiated by the linearly (vertically) polarized PALS iodine laser beam at the main frequency (1315 nm), the FWHM pulse duration of about 350 ps , and the energy up to 700 J. The laser radiation was incident normal to the target surface and focused to the spot with diameter of about 100 μm, thus providing the intensity up to $2 \cdot 10^{16}$ W/cm^2.
